# Smart antimicrobial efficacy employing pH-sensitive ZnO-doped diamond-like carbon coatings

**DOI:** 10.1038/s41598-019-53521-7

**Published:** 2019-11-21

**Authors:** Sascha Buchegger, Andrej Kamenac, Sven Fuchs, Rudolf Herrmann, Pia Houdek, Christian Gorzelanny, Andreas Obermeier, Stephan Heller, Rainer Burgkart, Bernd Stritzker, Achim Wixforth, Christoph Westerhausen

**Affiliations:** 1 Chair for Experimental Physics 1, University of Augsburg, Augsburg, 86159 Germany; 2 Department of Dermatology and Venereology, University Hospital Hamburg-Eppendorf, Hamburg, 20246 Germany; 3 Technical University of Munich, School of Medicine, Clinic of Orthopedics and Sportorthopedics, 81675 Munich, Germany; 4 Center for NanoScience (CeNS), Ludwig-Maximilians-Universität Munich, 80799 Munich, Germany; 5 Chair for Physiology, University of Augsburg, Augsburg, 86159 Germany

**Keywords:** Antimicrobials, Biomedical materials

## Abstract

One of the main challenges in endoprosthesis surgeries are implant-associated infections and aseptic-loosenings, caused by wear debris. To combat these problems, the requirements to surfaces of endoprostheses are wear-resistance, low cytotoxicity and antimicrobial efficacy. We here present antimicrobial coatings with a smart, adaptive release of metal ions in case of infection, based on ZnO-nanoparticles embedded in diamond-like carbon (DLC). The Zn^2+^ ion release of these coatings in aqueous environments reacts and adapts smartly on inflammations accompanied by acidosis. Moreover, we show that this increased ion release comes along with an increased toxicity to fibroblastic cells (L929) and bacteria (*Staphylococcus aureus subsp. aureus*, resistant to methicillin and oxacillin. (ATCC 43300, *MRSA*) and Staphylococcus epidermidis (ATCC 35984, *S. epidermidis*). Interestingly, the antimicrobial effect and the cytotoxicity of the coatings increase with a reduction of the pH value from 7.4 to 6.4, but not further to pH 5.4.

## Introduction

Due to the high number of orthopedic surgeries and the increasing number of antibiotic resistant bacteria, alternative approaches to diminish the number of clinical complications, like the possibility of smart reactions to infections, or antimicrobial effective surfaces, get more and more in focus. For example, smart hydrogels are described in literature^1–3^. These respond to pH changes with a change of absorbed water. Thus, the hydrogel swells extensively at intestinal pH values and therefore permits a higher drug release.

Furthermore, silica nanoparticles are under investigation for applications for controlled drug delivery. Those particles can be functionalized to control drug delivery through the opening of nanopores. This offers the possibility of pH-sensitive drug release^4^.

Another approach of pH-controlled drug delivery is a variety of pH-sensitive polymers^5^. A further approach to targeted drug release are magnetizable implants and functionalized magnetic carriers^6,7^. This approach does not react to an inflammation but enables targeted drug release directly at the affected implants by administering the functionalized drugs as a response to an inflammation. However, complexity, fragility and the absence of mechanical strength limit the field of applications of such materials for tribologically stressed surfaces.

In order to improve the mechanical strength and thus reduce wear, diamond-like carbon (DLC) coatings are often used in automotive industry, as machine parts or magnetic recording discs^8,9^. In addition, DLC often serves as a protective coating in biomedical devices or in endoprostheses^10–13^. While DLC itself is biocompatible, antimicrobial toxic effects can be achieved by doping of DLC coatings with Ag, Cu or V^14,15^

The antibacterial effect is due to the release of ions out of the incorporated metal in the biological medium and the subsequent uptake by cells and bacteria. Furthermore, ZnO nanoparticles possess antimicrobial effects towards bacteria like *Escherichia coli*, *Salmonella* or *Campylobacter jejuni*^16,17^. Moreover, it has been shown that ZnO nanoparticles exhibit a pH sensitive antimicrobial activity towards *E. coli* and *Staphylococcus aureus* in Glycerol^18^. In contrast to antibiotics, microbes were not able to develop resistances against these metal ions. Moreover, metal ions exhibit an extremely broad activity spectrum against a large range of different pathogens due to an unspecific mode of action.

**Figure 1:**
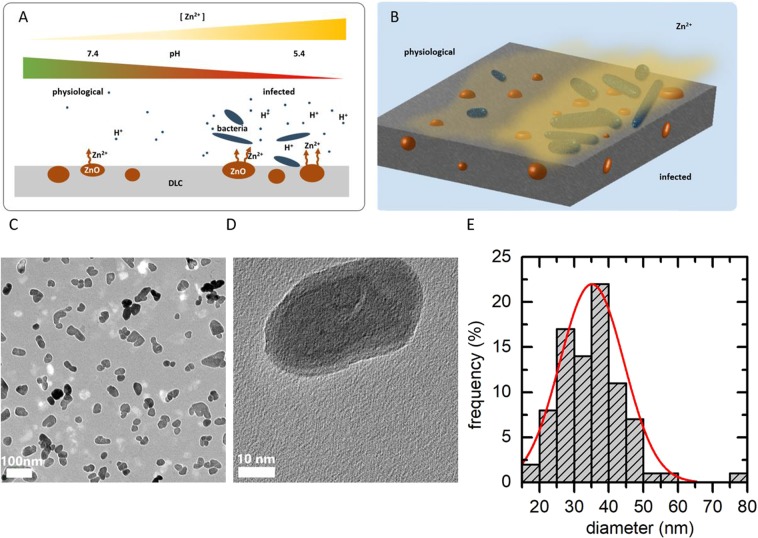
(**A,B**) Schematic illustration of the desired ion release behavior: A moderate ion release in case of physiological pH values and an increased ion release in case of acidic environment, which may be caused by bacterial infections. (**C,D**) TEM image of the ZnO nanoparticles (**E**) size distribution of the nanoparticles determined from TEM images.

We here incorporated ZnO nanoparticles in hard DLC coatings and measured the structural and mechanical properties like the fraction of sp^3^-hybridization as well as the morphology of the nanoparticles. We focused on the investigation of the effect of the pH of a buffer solution on the release of Zinc ions into this solution. Since the pH can drop from the physiological pH of 7.4 to acidic pH of 6 in case of inflammations, which is called acidosis^19,20^, we tested whether these functionalized surfaces can react in a smart way to infections by releasing Zinc ions depending on the actual needs (as illustrated in Fig. 1). In addition to the ion release measurements, we tested the toxicity of this coating to cells and bacteria in the relevant pH range.

## Results and Discussion

### Formation of Diamond-like carbon layers

Employing a plasma immersion ion implantation process to colloidal poly(vinylpyrrolidone) (PVP) films containing ZnO NPs, we prepare DLC-coatings and characterize these samples as described earlier^21^. Subsequently, we incubate the samples in aqueous solutions and measure their ion release kinetics.

**Figure 2:**
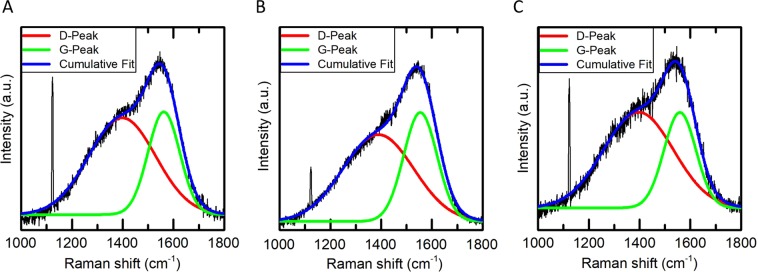
Raman spectroscopy measurements of DLC samples produced with a fluence of (**A**) 5 · 10^16^ cm^−2^, (**B**) 1 · 10^17^ cm^−2^ and **(C**) 2 · 10^17^ cm^−2^ showing a sp^3^-fraction of 30%, 33% and 28% respectively.

To investigate the role of the ion fluence during the DLC transformation for the ion release, we analyzed samples processed with a fluence of 5 · 10^16^ cm^−2^, 1 · 10^17^ cm^−2^ and 2 · 10^17^ cm^−2^ and the ion release of the according samples. The hybridization type of the carbon atoms was determined by Raman spectroscopy. The so-called D and G peaks in the spectra were fitted by Lorentzians and the intensity ratio of both modes, I(D)/I(G), was calculated. As can be seen from Fig. 2, the lowest I(D)/I(G) ratio of 1.69 can be achieved by applying the fluence of 1 · 10^17^ cm^−2^. Doubling the fluence to 2 · 10^17^ cm^−2^ or reducing it by a factor of two to 5 · 10^16^ cm^−2^ leads to an increased ratio of 1.98, and 2.18, respectively. The shift of the G-Peak shows the same trend with the lowest wave number of 1554 cm^−1^ for the fluence that was shown to be optimal for this process^21^ and higher wavenumbers of 1562 cm^−1^ and 1559 cm^−1^ for the halved and the doubled fluence. Both parameters are indicators for the sp^3^ fraction^22^. By comparison of the measured parameters with literature values^9^, we estimated a sp^3^-fraction of 33% for the sample with an optimal fluence of 1 · 10^17^ cm^−2^. The fraction of sp^3^-hybridized atoms decreases to 28% by applying twice the optimal fluence and shows 30% in case of half the optimal fluence. The former effect is conform to graphitization as has been shown in previous studies^23^. A fluence lower than the optimal fluence, on the other hand, does lead to incomplete cross-linking and densification of the polymer precursor layer and therefore the latter samples likewise exhibit a lower sp^3^-fraction. Nevertheless, in summary, all samples exhibit typical values for a-C:H and are thus suitable for various applications.

**Figure 3:**
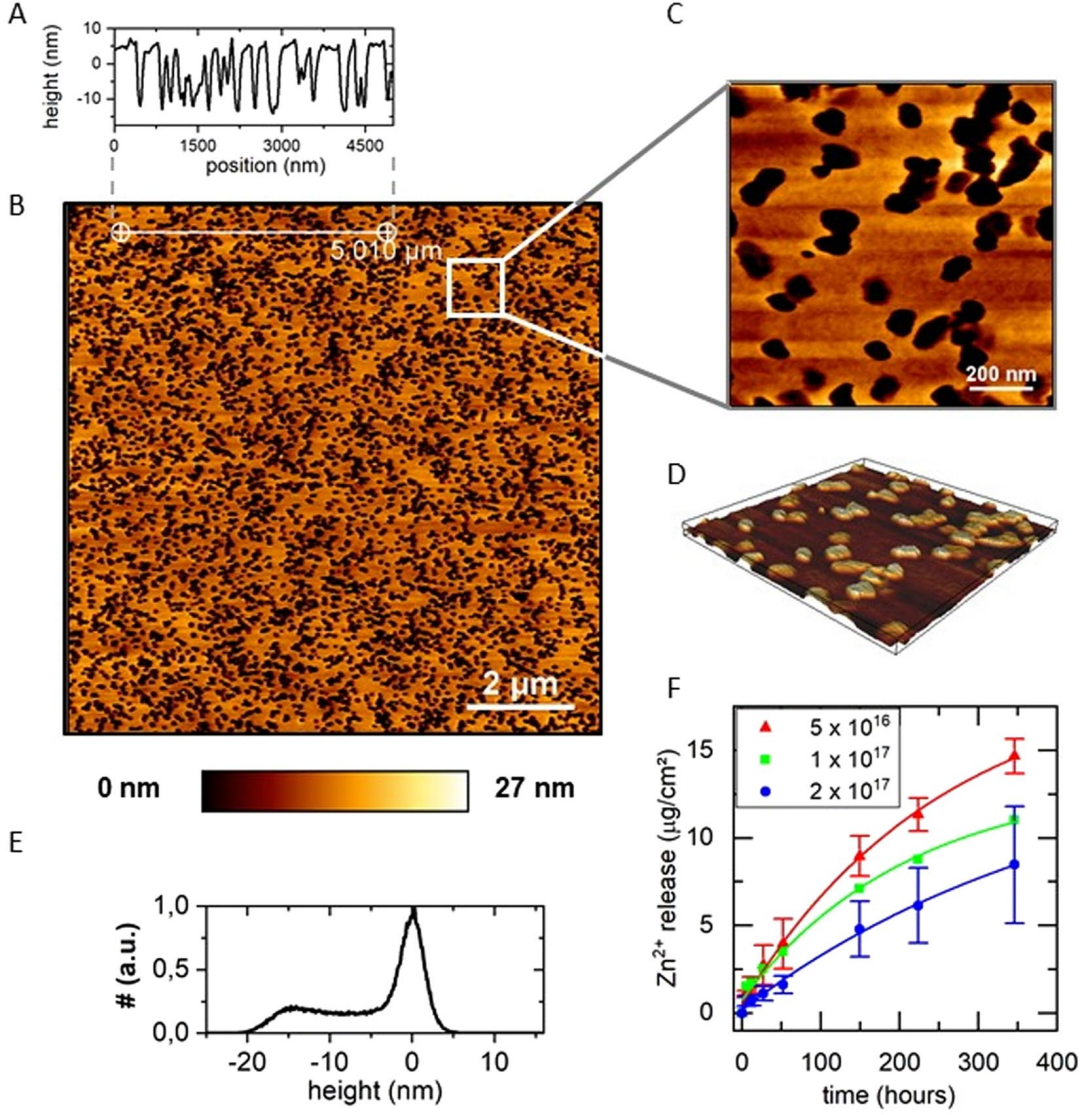
Surface topography and ion release kinetics (**A**) Height profile and (**B**) AFM micrograph of a ZnO-DLC surface after ion release. (**C**) Zoom in in B. (**D**) inverted 3D image of C. (**E**) Height histogram of B. (**F**) Ion release from ZnO-DLC coatings for different fluences during DLC-transformation process.

Figure 3 shows the surface topography and the Zn ion release kinetics for these different fluences during the DLC transformation step. Obviously, after the dissolution of the ZnO-NPs by incubating the samples in aqueous solution, holes in the size of the used NPs remain in the coating. This increased (and by variation of the NP-size) tunable roughness could be advantageous for osteoblast adhesion, e.g. in the case of a coated prosthesis shaft. As can be seen, the cumulated Zn^2+^ release decreases continuously with increasing fluence. This may not be explained exclusively by the fraction of sp^3^ hybridized carbon atoms and the density respectively. It may be rather explained by a progressive drop in thickness with increasing fluence. The thickness d = 126 nm of the polymer precursor layer drops to d = 79 nm for a fluence of 5 · 10^16^ cm^−2^ and to d = 67 nm and d = 50 nm for fluences of 1 · 10^17^ cm^−2^ and 2 · 10^17^ cm^−2^ respectively, caused by sputtering on the one hand and densification on the other hand. By normalizing the ion release kinetics to the final thickness of the DLC transformed coating, all tested samples show the same behavior within the error bars. This indicates that within the range of a-C:H the fluence has a minor impact on the ion release kinetics. It is rather dominated by the film thickness and accordingly to the total amount of ZnO nanoparticles in the film. This is in accordance with previous studies, in which the amount of released ions is tuned by adapting the nanoparticle concentration in the coating^21^. Thus, it is possible to tune the ion release kinetics by adjusting both the nanoparticle concentration in the coating as well as the fluence during DLC transformation. However, while the former effect is much simpler to implement, the latter may not be neglected, if for other reasons the fluence e.g. is used to fine tune mechanical properties of the coating.

### pH-dependent zinc ion release

Aiming towards a smart antibacterial efficacy the Zn ion release in solutions of various pH between 5.4 and 9.4 was determined. As stated above, in case of inflammations, the pH of the environment of such a prosthesis acidotically can drop strongly below pH = 7.4.

**Figure 4:**
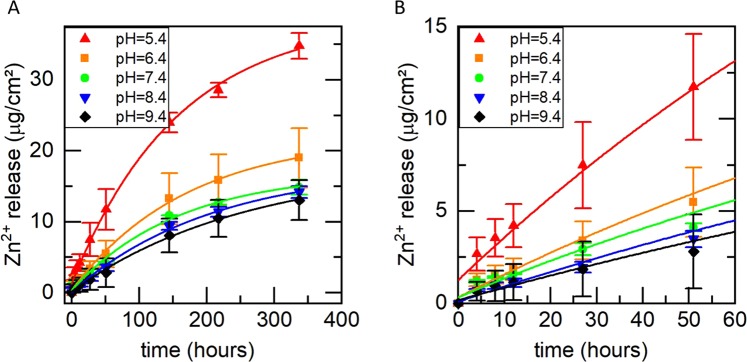
(**A**) Ion release kinetics of ZnO-DLC-coatings in dependence of the pH (**B**) Zoom-in of A) for the initial 60 hours.

As can be seen from Fig. 4, the ion release within the first 336 hours ranges between 13 µg/cm² and 35 µg/cm² and depends non-linearly on the pH of the buffer conditions. While the release is comparable for physiological pH of 7.4 and above, it is strongly increased with decreasing pH. At a pH of 6.4, the amount of released Zn^2+^ within the first two weeks is increased by about 30% compared to pH 7.4. A further decrease of the pH to 5.4 leads to an increase of released Zn^2+^ of even 130%.

To describe our experimental findings, the data were fitted with an exponential function $$A(t)=A[1-$$$$\exp (\,-\,\frac{t}{{t}_{1}})]+{\rm{C}}$$, where A represents the overall amount of Zn and t_1_ represents the time constant of the exponential Zn ion release. The results are presented in Table 1.

**Table 1:** Parameters of the fit function for the data shown in Fig. 5.

pH	Total release A	Time constant t_1_
9.4	17.7 ± 1.6	256.9 ± 40.7
8.4	17.6 ± 1.1	208.4 ± 26.7
7.4	16.6 ± 1.0	157.2 ± 23.9
6.4	21.7 ± 0.9	169.6 ± 16.7
5.4	37.5 ± 1.5	157.3 ± 15.5

The parameters A of the fit functions differ for the different pH values, although all samples are prepared simultaneously in one step and therefore should contain the same amount of ZnO. This physically unexpected result is presumably caused by the fact that after two weeks, only a fractional amount of the ZnO is released and the ion release process is far away from its endpoint. Nevertheless, within the clinically relevant time period of two weeks, the ion release kinetics are described very well by the presented fit functions.

**Figure 5:**
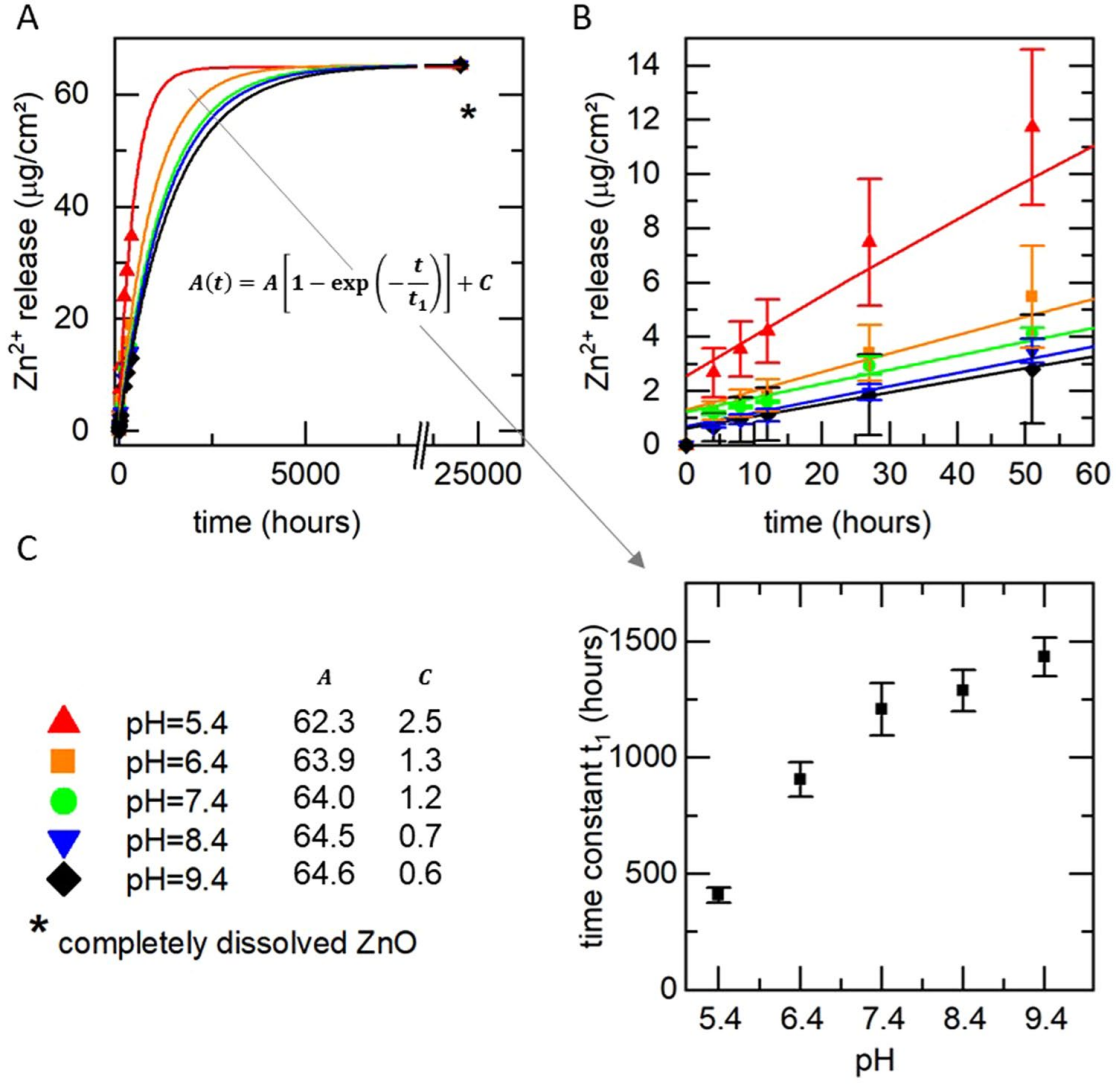
(**A**) Fit curves of the measured Zn^2+^ release. An arbitrary end point was chosen at t_end_ = 1,000 days, which is sufficient high for converging of the fit function. (**B**) Zoom in to (**A**) for the initial 60 hours (**C**) Fit parameter (**A,C**) and time constant t_1_ of the Zn^2+^ release in dependency of the pH.

To model the ion release, however, in a physical meaningful way, we added an artificial final point to the measurement data, as shown in Fig. 5. For this purpose, we measured the total amount of released Zn^2+^ by immersing the samples for two weeks in 0.1 molar HCl and subsequently measuring the concentration of released Zinc in the solution. The point of time of this additional endpoint was arbitrarily set to 1,000 days, which is high enough to allow all fit functions to converge. By inclusion of this final point, the parameter A of the fit function converges for all samples to 63.5 ± 1.5 µg/cm^2^.

The time constant t_1_ obtained from these model fits in dependency of the pH is presented in Fig. 5B. As can be clearly seen, the ion release kinetics are accelerated in the acidic regime whereas it only changes slightly for pH values of 7.4 and higher. Within the acidic regime, the time constant t_1_ is continuously decreasing with decreasing pH. It drops from 1,208 hours at pH 7.4 to 906 hours and 407 hours at pH values of 6.4 and 5.4, respectively. This means a decrease of the time constant t_1_ of 25% and 66% respectively in comparison to the physiological pH of 7.4. This represents the desired behavior, which should exhibit a moderate ion release in case of physiological pH and a boost of released Zn^2+^ in case of a drop in pH, which may be caused by bacteria.

Our findings qualitatively correspond to studies described in literature, in which an increased toxicity of ZnO nanoparticles in glycerol was observed^18^. Thus, implants coated with ZnO-NPs containing DLC can react in an intelligent-like and adaptive manner to inflammation-caused acidosis and provide the antimicrobial agent depending on demand.

### Cytotoxicity to fibroblastic cells

**Figure 6:**
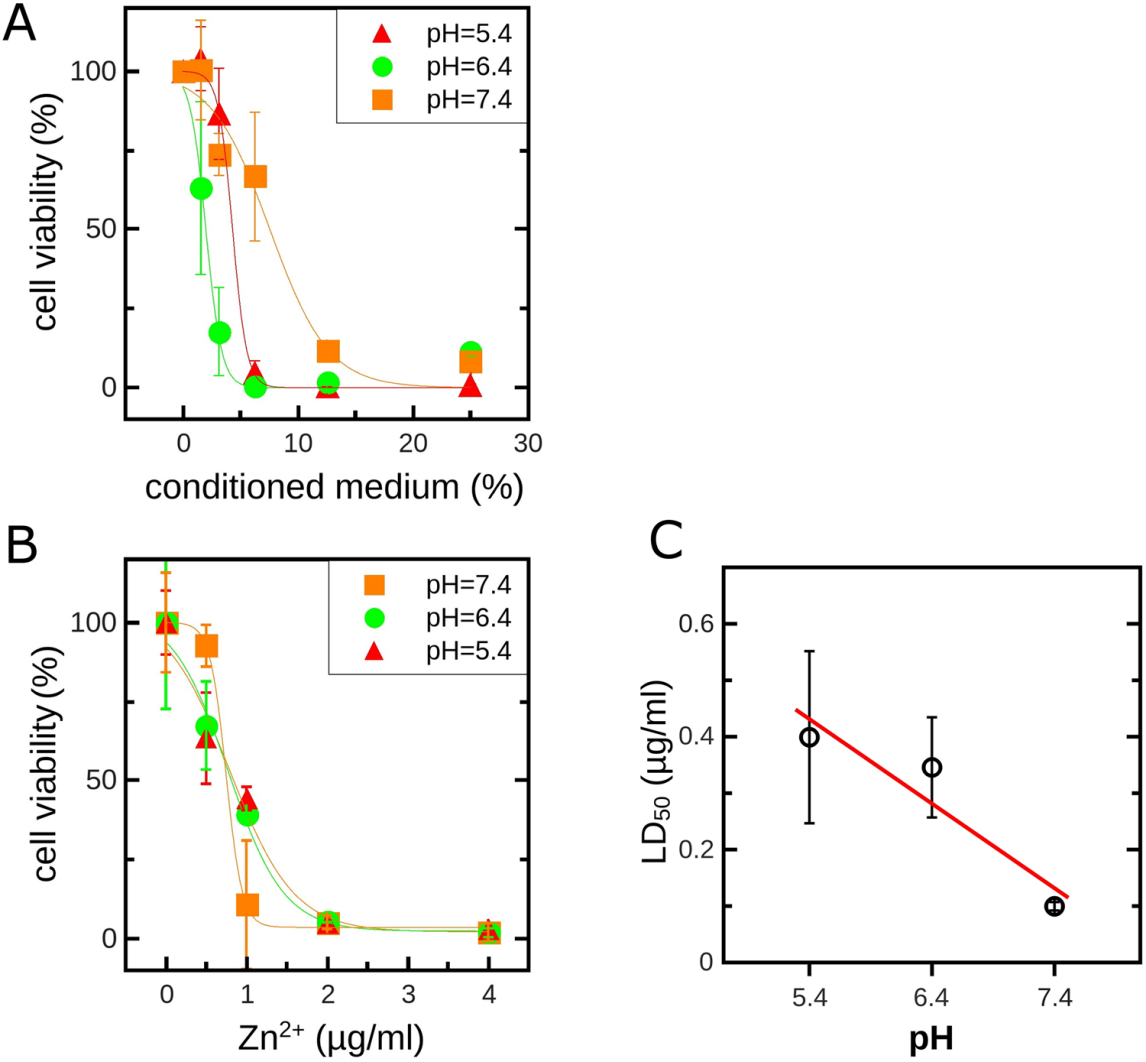
Metabolic activity of cells (type L929) treated with different fractions of implant-conditioned Hank’s balanced salt solution (**A**) or defined concentrations of ZnCl_2_ (**B**). LD50 values derived from (**B**) indicate a roughly linear correlation between the pH of the buffer and the toxicity of ZnCl_2_.

Complementary to the measurement of the ion release kinetics, the cell viability on our coatings was tested *in vitro* (see Fig. 6). Implant specimens without cells were incubated for 24 h in Hank’s balanced salt solution (HBSS) adjusted to pH 5.4, 6.4 or 7.4 respectively. Different fractions of this conditioned medium were applied to the fibroblastic cell line L929. The presented data represent the mean and the standard deviation of three independent experiments.

Figure 6A shows the impact of the pH-dependent liberation of Zn^2+^ on the cell viability. With increasing fractions of the conditioned medium cell viability was reduced. However, the decay of the cell viability was steepest at pH 6.4 suggesting the highest sensitivity for this pH. This is partially in line with the pH-dependent release of Zn^2+^ from the implant coatings and suggest increased levels of ions and thus increased toxicity at pH 6.4 in comparison to pH 7.4. However, the supposed higher levels of Zn^2+^ at pH 5.4 (compare Figs 4 and 5) did not correlate with a further reduced cell viability. Indeed, Zn^2+^ homeostasis in mammalian cell is sensitive to the pH^24–27^ and the potentially harmful binding of Zn^2+^ to amino acids such as cysteines is attenuated at lower pH values^28^. Therefore, to further prove that not only the release of Zn^2+^ from the implant coatings is pH-dependent but also the zinc toxicity, we performed additional experiments with defined concentration of ZnCl_2_ salt added to the pH-adjusted HBSS. Figure 6C shows the ZnCl_2_ dose- and pH-dependent change of the cellular viability. Deviated LD50 values indicate a roughly linear correlation between the pH of the medium and the toxicity of ZnCl_2_ (Fig. 6C) confirming that at pH 5.4 L929 cells are more protected against Zn^2+^ than at pH 6.4 or 7.4.

### Bacterial adhesion assay

*Staphylococcus aureus subsp. aureus*, resistant to methicillin and oxacillin*. (ATCC 43300, MRSA)* and *Staphylococcus epidermidis (ATCC 35984, S. epidermidis)*, reported to produce polysaccharide adhesion^29^ were used as test strains for *in vitro* experiments. These species are clinically relevant pathogens and cause implant-associated infections. We measured colony forming units (cfu) in bacterial adhesion assays, in the supernatant, as well as the size of antimicrobial inhibition zones.

**Figure 7:**
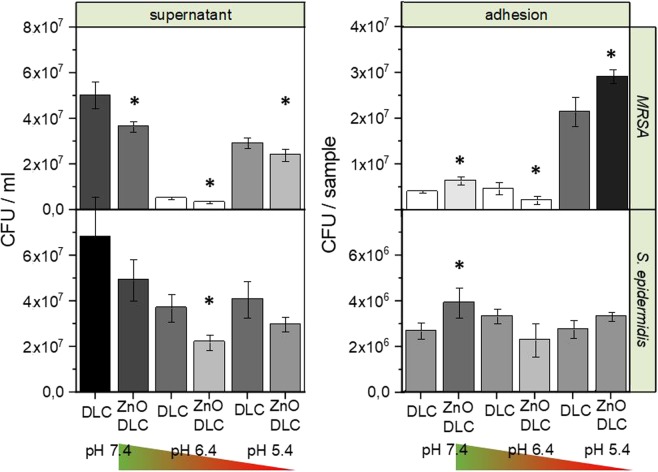
(Left) cfu values for *MRSA* and *S. epidermis* in the supernatant and (Right) adhered on the surface. Each cfu value is the average of at least 4 samples.

The bacterial adhesion assay shows the highest bacterial reduction by ZnO-DLC-coatings referred to the DLC controls at pH 6.4. Bacterial reduction at varied pH-values for *MRSA* and (*S. epidermidis)* in the supernatant reached (pH 7.4: −27% (−28%), pH 6.4: −34% (−40%), pH 5.4: −18% (−27%)) as well as on the sample surfaces (pH 7.4: +58% (+45%), pH 6.4: −53% (−31%), pH 5.4: +16% (+20%)). A significant reduction was detected for *MRSA* for each pH value. In contrast, a significant reduction could only be shown for *S. epidermidis* adhered on sample surfaces at pH 7.4 and in the supernatants of pH 6.4 media. The bacterial count numbers for each test and pH value for both test strains can be seen in Fig. 7.

### Agar diffusion assay

**Figure 8:**
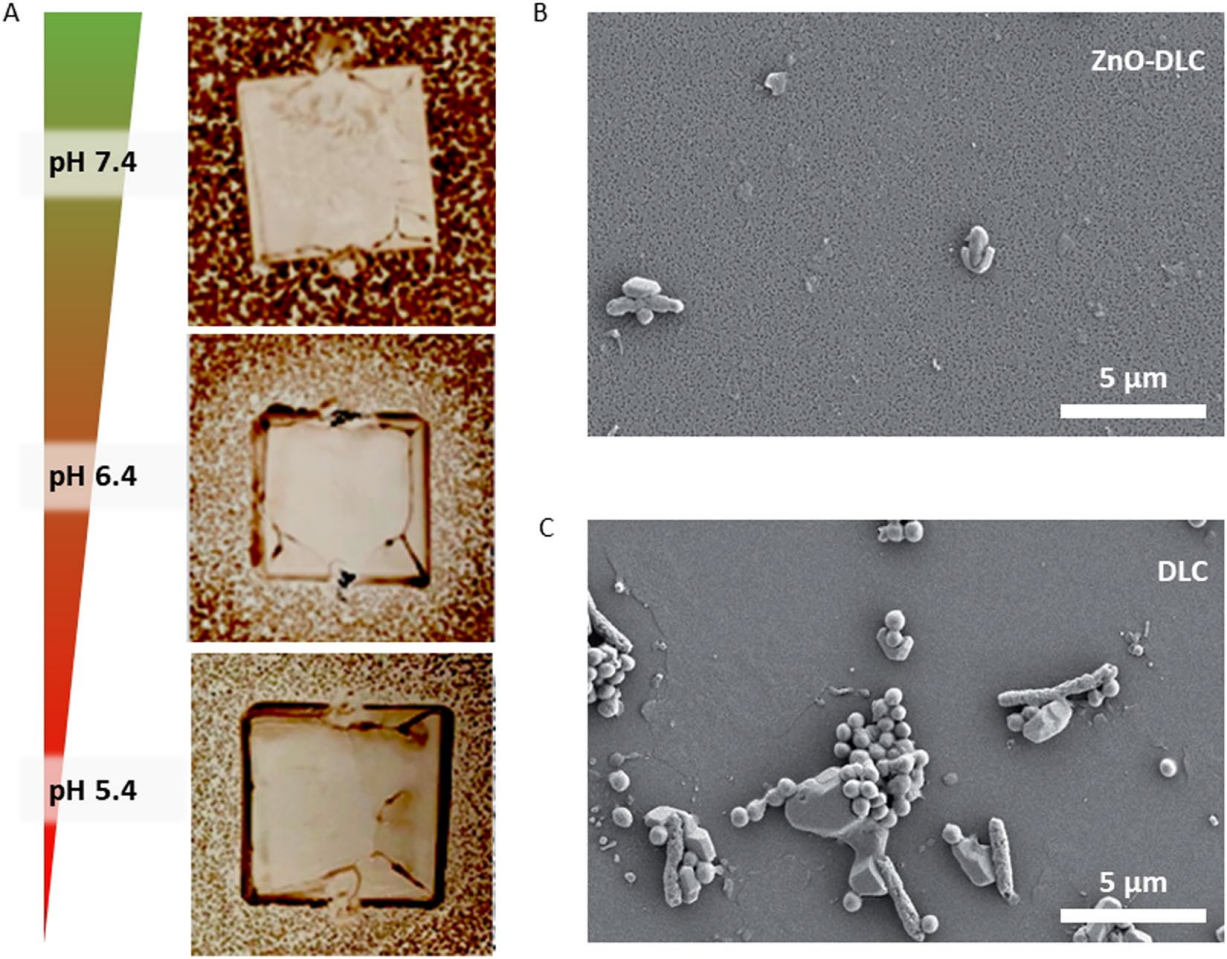
(**A**) Antimicrobial inhibition zones of 9 × 9 mm^2^ DLC-ZnO-samples against *MRSA* on MHA as function of pH (**B,C**) Scanning electron micrographs of a ZnO-DLC sample and a DLC reference sample after incubation with *MRSA* at pH 6.4.

Figure 8 shows the antimicrobial inhibition zones of 9 mm DLC-ZnO-samples against *MRSA*. Mean inhibition zones of three replicates showed a lateral length of 9.5 mm (pH 7.4), 13.0 mm (pH 6.4) and 10.5 mm (pH 5.4).

Although it was shown that the release of ZnO-NP is highest at pH 5.4, the adhesion assay revealed that for the tested bacterial strains the antimicrobial efficacy of these nanoparticles is greatest at pH 6.4. Only at pH 6.4 the number of bacteria could be reduced both in the supernatant, as a result of ion release, as well as on surfaces, because of contact inhibitions.

In addition, inhibition zones generated via an agar diffusion assay showed an pH-dependent impact on the antimicrobial effect of the diffusing Zinc ions against the Methicillin-resistant Staphylococcus aureus subsp. aureus (*MRSA*). The results of the agar diffusion assay confirmed the results of the adhesion assay. The largest inhibition zones were found for adaptive implant surface modifications with ZnO-NP at a pH of 6.4. Therefore, both microbial tests led to the conclusion that the antimicrobial effect of the ZnO-NP increases with a reduction of the pH value from 7.4 to 6.4, but not further to pH 5.4.

Based on these data, it must be assumed that there obviously exist other pH-dependent effects which influence the interaction of zinc ions and pathogens and thus also the antimicrobial efficacy. Moreover, it indicates that there is no linear relationship between pH and antimicrobial efficacy. Presumably, a reason of pH-dependent charge-modifications of Zinc ions. This hypothesis and further aspects have still to be investigated basically, in order to improve the antimicrobial efficacy for future clinical applications.

Finally, we would like to address a controversy, as the present surface modification not only causes the desired antimicrobial activity, but also a high cytotoxicity. This is due to the broad unspecific activity spectrum of metal ions in general. However, the effect here still is beneficial as ZnO-NP incorporated DLC is characterized by a fast and strong release of zinc ions to ensure the efficient and local elimination of pathogenic microbes during the surgery in a small volume. Postoperative, the cytotoxic zinc ion concentration will be consecutively diluted in the whole body and incorporated into the physiological zinc metabolism. After the zinc ions are released from the implant and their concentration approaches to a non-cytotoxic level, host cells can settle on the surface and start to integrate the implant into the tissue.

## Conclusion

DLC-coatings with embedded nanoparticles were successfully produced by ion induced diamond-like carbon transformation of a polymer. The release of Zn^2+^ was measured under various conditions. We obtained release kinetics, which are almost independent of the fluence used in the DLC-transformation. The release kinetics are rather dominated by the concentration of ZnO nanoparticles in the DLC film on the one hand and by the pH of the surrounding medium on the other hand. The latter was demonstrated by ion release measurements, where we found that the release of Zn^2+^ is accelerated by 30% from pH 7.4 to pH 6.4 and by even 130% to pH 5.4 while it is almost constant in the basic regime. The ion release kinetics and the toxicity of these adaptive coatings was confirmed in biological control experiments. We found that their cytotoxicity is strongly pH dependent and shows the same trend as the ion release kinetics.

However, the antimicrobial effect against *MRSA* and *S. epidermis* of the coatings increases with a reduction of the pH value from 7.4 to 6.4, but not further to pH 5.4. In line with these findings for bacteria, the LD50 values of the L929 cells for ZnCl_2_ indicate that a low pH L929 cells are more protected against Zn^2+^ than at pH 6.4 or 7.4.

In future studies, the concentration of ZnO nanoparticles in the DLC coating should be adapted, which can be done easily to find a good balance between sufficient cell integration at physiological pH and high enough toxicity at acidic pH. Nevertheless, these novel and powerful coatings offer a great potential in orthopedic implant design since they adapt smartly to bacterial acidosis and supply the antimicrobial agent on time as requested, as we showed here for the first time.

## Experimental section

### Surface modifications and characterization of ZnO nanoparticles in DLC

Elliptical-shaped zinc nanoparticles (ZnO-NPs) with a mean diameter of 35 ± 15 nm were synthesized using a method described in the literature^30^. We prepared a dispersion containing 500 mg of ZnO-NPs and 5.0 g of PVP (MW 55,000) in 145 ml dry ethanol. Cylindrical samples (Ø: 10 mm, thickness: 2 mm) of a titanium alloy (Ti6Al4V), and square samples (9 × 9 mm², thickness 1 mm) of silicon were used as the substrate material of our implant phantoms. The substrates then were mounted on a stainless-steel plate substrate holder with adhesive carbon tape and then finally dip-coated with the ZnO-PVP-dispersion. The film thickness was controlled (using a profilometer) to 126 + −5 nm by adjusting the retraction speed to 1.4 mm/s. Subsequently, this NP containing polymer matrix was transformed to DLC by ion implantation in a plasma immersion ion implantation step. Cross-linking, densification and a rearrangement of atoms and bonds due to the ion bombardment finally led to the formation of an about 50 nm thick DLC-layer. In this step, we used a process gas mixture containing 47.7% neon, 32.7% argon and 19.6% methane at a pressure of 3 · 10^−3^ mbar. Then, 800 W of microwave power was applied to generate a plasma in an electron cyclotron resonance (ECR) plasma source. The ions within the plasma were accelerated towards the sample’s surface by applying high negative voltage pulses of −20 kV with a repetition rate of 200 Hz and a pulse width of 5 µs. If not explicitly mentioned the process time of all samples was 73 min, resulting in an implanted fluence of about 1 · 10^17^ cm^−2^, being optimal for the DLC transformation of PVP, as shown in prior studies^31^. In addition to the optimal fluence of 1 · 10^17^ cm^−2^, also fluences of 5 · 10^16^ cm^−2^ and 2 · 10^17^ cm^−2^ were used to investigate the influence of the DLC matrix on the ion release kinetics.

To characterize the coating by determining the fraction of sp^3^ hybridized carbon atoms, micro Raman measurements were carried out by using the 514 nm line of an Ar-Ion-Laser at 1 mW. The laser spot was focused on the sample’s surface using a 100x microscope objective. The spectra of the scattered light were recorded with a CCD spectrophotometer. To estimate the fraction of sp^3^ hybridizations, the D-Peak as well as the G-Peak were fitted with a Gaussian and the ratio of accumulated intensity of both peaks was compared with the experimental results reported in the literature^9^. Nano hardness measurements were performed using an UNAT nano indenter (Asmec, Rossendorf, Germany). A force ramp with a maximum force of 0.6 mN was applied to the Berkovich type measuring tip. We used the QCSM mode, whereby an additional force vibration was modulated on top of the force ramp to investigate the hardness depth-dependent. Size and morphology of the nanoparticles shown in Fig. 1 were characterized by transmission electron microscopy using a JEM 2100 F (JEOL Ltd., Tokyo, Japan) microscope.

### Zinc ion release kinetics

To study the zinc ion release of the functionalized surfaces, the samples´ edge and bottom were painted with rust inhibitor (Hammerite, AkzoNobel N.V., Netherlands) to firstly, start with a defined surface area, secondly to avoid an undesired release of Zn ions from the shell or from the sample´s bottom, where the polymer is not necessarily transformed to DLC. Two samples were embedded in each glass beaker and subsequently exposed to 10 ml of a buffer solution. To avoid precipitation of zinc phosphate^32^, we used phosphate-free PIPES buffer of pH values between 5.4 and 9.4. After defined time periods, the supernatant was exchanged and the concentration of Zn was measured employing Inductively Coupled Plasma Optical Emission Spectroscopy (ICP-OES) using a Vista MPX (Varian, Palo Alto, USA).

### Viability of cells on ZnO-DLC surfaces

Additional to the ion release measurements, the viability of fibroblastic cells (L929) upon exposure of Zn^2+^ released from the surface of the implant was tested by WST (water soluble tetrazolium)−1-assays (Roche). The test is based on the absorbance of a formazan dye formed by mitochondrial dehydrogenase from WST-1. For this test, 710.500 cells of the L929 cells were seeded into the well of a 96 well plate and cultivated for 24 h in culture medium (DMEM supplemented with 10% fetal calf serum, 1% L-glutamine and 1% penicillin/streptomycin) at 37 °C in humidified air containing 5% CO_2_. In parallel, implant specimen were immersed in 8 ml, 16 ml and 32 ml Hank’s balanced salt solution (HBSS) with pH values adjusted to 5.4, 6.4 or 7.4. After 24 h, the conditioned HBSS was collected and 100 µl were added to the cells in substitution to the cell culture medium. After another 24 h of culturing, the buffer solution was replaced by 100 µl serum free DMEM, containing 10% (v/v) of the WST-1 reagent. The formation of the WST-1 was recorded after 2 h with a photometer by measuring the extinction of light at 450 nm. The metabolic activity of the cells scales with the extinction. As reference samples, L929 were either treated with 0.1% (v/v) triton X-100 in serum free DMEM (0% cell viability) or pH adjusted HBSS containing no Zn^2+^ (100% cell viability). Additional control experiments, using L929 cells treated with a range of different ZnCl_2_ (Sigma Aldrich) concentrations (4.0, 2.0, 1.0, 0.5 µg/ml) in HBSS with a pH of 5.4, 6.4 or 7.4.

### Bacterial adhesion assay

Both bacteria were pre-cultured on Columbia Agar plates (CBA, BD Diagnostics, Heidelberg, Germany). The disk-shaped samples to be examined were fixed with paraffin under aseptic conditions in 24 well plates. In the beginning, a home-made carbonate buffer including 1% Mueller-Hinton Broth (MHB) was adjusted to pH values 7.4, 6.4 and 5.4 (±0.1) at room temperature using HCl (1 M) titration, adjusted with a pH meter (PB-11, Sartorius, Göttingen, Germany). The bacterial suspensions were adjusted to an optical density of OD600 (0.1 ± 0.02) and diluted 1:4000 in MHB, resulting in approximately 10^4^ cfu/ml. Titan samples modified with DLC (control group) and DLC + ZnO-NP (test group) were inoculated each with 1 ml bacterial suspension (10^4^ cfu/ml) at different pH-values and incubated at 37 °C for 24 hours using an 5%-CO_2_-incubator. To investigate the bacterial numbers in the supernatants, 500 µl were taken and diluted (1:1) with a neutralization solution to stop Zinc ion activities (consists of 1.0 g sodium thioglycolate, 1.46 g sodium thiosulfate and 1 liter sterile Aqua dest.). Then, dilution series were made and 100 µl from each sample were plated on Agar. Colony forming units were counted after incubation overnight. On the other hand, to determine viable adhered bacterial numbers, samples were taken out of the well plates, washed in sterile phosphate buffered saline (PBS) and transferred in tubes, containing 1.5 ml PBS and 0.5 ml neutralization solution. Samples were then vortexed (10 s), sonicated (5 min. @35 kHz) and again vortexed (10 s) to release adhered bacteria from surfaces in the surrounding media. The gained bacterial suspension was diluted and plated on Agar for each sample. After incubation overnight, cfu were counted.

### Agar diffusion assay

For carrying out the agar diffusion assays, home-made Mueller Hinton Agar plates (MHA) with adjusted pH-values at 7.4; 6.4 and 5.4 were used. Agar media were prepared by adding HCl (1 M) 0%; 0.3% and 0.4%, respectively. Subsequently, a bacterial suspension with a density of OD600 0.1 was used to inoculate MHA plates, then controls (DLC without ZnO-NP) and samples (DLC with ZnO-NP) were set on MHA plates and incubated at 37 °C overnight. On the following day, the size of each resulting inhibition zone was measured by a caliper gauge in millimeter.
